# On the importance of structural equivalence in temporal networks for epidemic forecasting

**DOI:** 10.1038/s41598-023-28126-w

**Published:** 2023-01-17

**Authors:** Pauline Kister, Leonardo Tonetto

**Affiliations:** Technical University of Munich, Munich, Germany

**Keywords:** Computational science, Scientific data

## Abstract

Understanding how a disease spreads in a population is a first step to preparing for future epidemics, and machine learning models are a useful tool to analyze the spreading process of infectious diseases. For effective predictions of these spreading processes, node embeddings are used to encode networks based on the similarity between nodes into feature vectors, i.e., higher dimensional representations of human contacts. In this work, we evaluated the impact of *homophily* and *structural equivalence* on node2vec embedding for disease spread prediction by testing them on real world temporal human contact networks. Our results show that structural equivalence is a useful indicator for the infection status of a person. Embeddings that are balanced towards the preservation of structural equivalence performed better than those that focus on the preservation of homophily, with an average improvement of 0.1042 in the f1-score (95% CI 0.051 to 0.157). This indicates that structurally equivalent nodes behave similarly during an epidemic (e.g., expected time of a disease onset). This observation could greatly improve predictions of future epidemics where only partial information about contacts is known, thereby helping determine the risk of infection for different groups in the population.

## Introduction

The recent outbreak of COVID-19 showed the importance of knowing the process of how a virus spreads in a population^1^. If the exact path a virus takes from person to person is known, better containment strategies, vaccination plans and preparations for future epidemics could be developed^6, 13, 25, 28^. However, a process that might seem simple – one person infects the next – can quickly become complex in a network such as human society. The characteristic topology of a human contact network, including the scale-free distribution of node degrees and the formation of clusters, has an impact on the path a disease takes through these networks^4, 14, 16^. Furthermore, recent advances in human mobility and network epidemiology research allow us to expand our understanding of such complex problems. That is, if the temporal contacts network and attributes of the disease being studied are known, such as infection and recovery rate, it is possible to simulate the trajectory of epidemic outbreaks. Furthermore, machine learning models can facilitate the prediction of the remaining nodes, even if the disease attributes are not known and only partial information about a few infected nodes exists. One limitation of this approach is that most machine learning methods expect a set of feature vectors as input instead of a graph. That is, models are trained and validated using expected outcomes and not a representation of how nodes are connected over time. As a currently widely used solution, these learning methods rely on node embedding to convert the contact network into a set of feature vectors. The task of the node embedding is to represent each node in the network as a low-dimensional feature vector while preserving the structure of the graph as much as possible. This means that if two nodes are structurally similar, their resulting feature vectors are close to each other in the embedding space. In this way, information about node similarity can be preserved in this new representation. How good an embedding is depends on the context and the task it is used for. In the context of disease spreading, the embedding of a contact network should preserve all the information necessary for predicting *who* is infected, and *when*.

Common embedding methods used for disease spreading are based on the fact that a disease spreads from person to person, between nodes who are connected for sharing some kind of similarity, which corresponds to *homophily* in a network. Another relevant concept in network modelling is *structural equivalence*, where nodes with the same position in the structure of the network are infected at the same time. In real world networks, both of these notions of similarity appear at the same time and their influence for networks anaysis varies for different kinds of networks^10^.

A popular embedding method is node2vec^10^, an algorithm based on random walks. To discover and encode a graph’s topology, node2vec conducts random walks through the graph. These random walks can be either biased towards *inward* exploration while preserving homophily in the network, or towards *outward* exploration preserving structural equivalence. In this work, we used node2vec with inward, neutral and outward oriented random walks and compared predictions about the state of each node made by a logistic regression algorithm. This analysis reveals that the best predictions can be made with outward oriented random walks, which suggests that structural equivalent nodes show similar behaviour during the infection process, that is, better predictive models are obtained when structural equivalence is primed. In turn, these observations suggest that two or more structurally equivalent nodes in a network may be at similar risks of infection even if they are not directly connected, potentially helping contact tracing efforts during an infectious disease outbreak.

The influence of homophily and structural equivalence in a community has been observed by sociologists in many different contexts. In sociology, homophily in a network corresponds to *cohesion* or *strong ties* in society, while structural equivalence corresponds to *weak ties*. In some contexts, weak ties are more important than strong ties^9^, which speaks for the importance of structural networks in human contact networks. In more recent studies, considering weak ties was helpful in different sociological contexts, such as the influence of indirect contacts on decision making^29^, or the dismantling of organized crime^15^. In a more graph theoretical approach, researchers explored different methods of clustering people by their roles, relying on the fact that structurally equivalent nodes fulfill the same role in society^19^.

While most node embedding methods focus on homophily, some have been developed to preserve only structural equivalence. The method proposed by Wang *et al.*^27^ has a precise mathematical approach, while struc2vec^5^ is based on random walks similarly to node2vec. Another popular embedding method uses the recursive nature of structural equivalence to create an embedding, as two nodes are considered structurally equivalent if their neighbors are structurally equivalent^26^.

While reviewing node embedding methods, Junchen *et al.* confirmed that node2vec embeddings perform best against other methods at preserving local structure^12^, whereas Schliski *et al.* included notes of caution by testing node2vec with different hyperparameter settings and concluded that node2vec does not preserve structural equivalence well, even with outward orientated hyperparameters^22^. However, these results should not affect our analysis as the authors focused at global structural equivalence, and we chose node2vec as it tries to achieve a balance between local structural equivalence and homophily.

## Results

We observe that embeddings with outward exploration result in better predictions of epidemic dynamics than embeddings with inward exploration. That is, structural similarity has a stronger predictive power than the exact nearby neighbors of a node, or a node’s role in a network may be more relevant than who their peers are for a disease outbreak outcome.

Our features used for prediction may consider either nodes with common neighbors to be similar, obtained through *inward* exploration, or nodes surrounded by an equivalent structure of neighbors to be similar, obtained through *outward* exploration (see “Methods” section). Furthermore, we evaluate our approach using the f1-score, which balances the accuracy of true and false predictions of positive and negative cases in all time steps of the outbreak considered (see “Evaluation Metrics” section). We run 50 simulations for each combination of dataset and SIR parameters, and report the averages and confidence intervals of the results.

**Figure 1:**
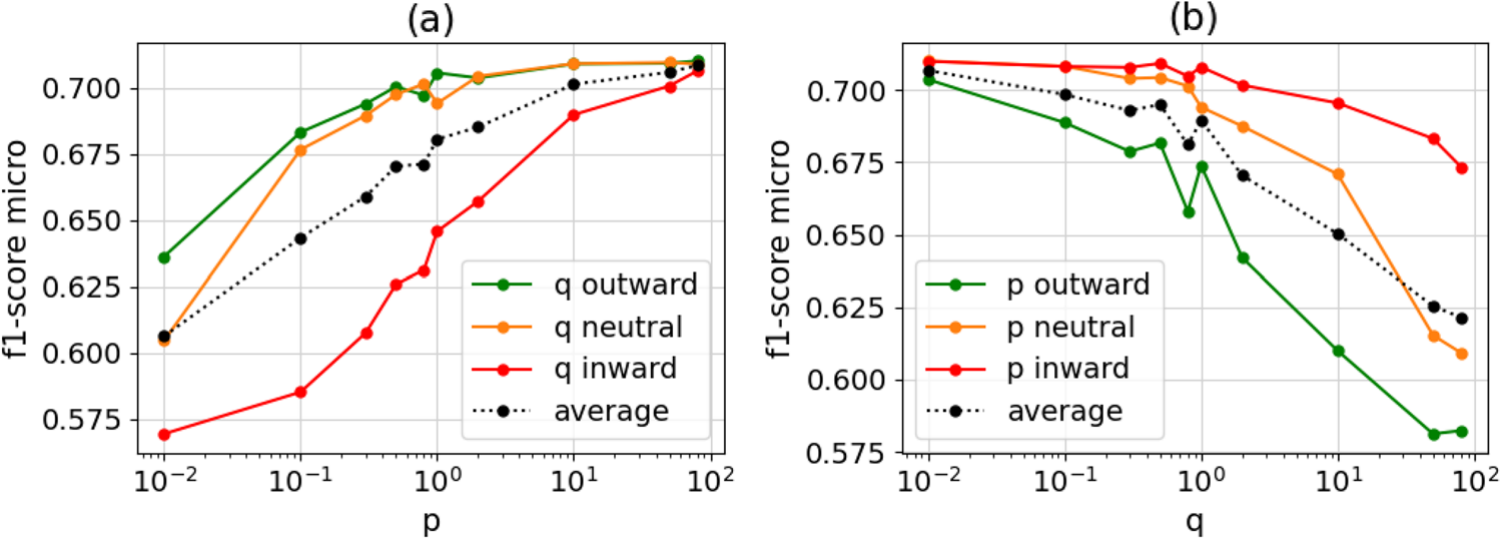
Comparison of inward, outward and neutral parameters.

We performed simulations on a total of 24 networks, with 6 of them derived from publicly available datasets, which are described in “Methods” section. By prioritizing *outward* explorations (*i.e.*, with a higher return parameter p, or a lower in-out parameter q), we obtain a better f1-score, as depicted in Fig. 1a and b. From our simulations, the best prediction results were achieved at *p* = 50 and q = 0.01. In comparison to the neutral values, outward exploration only achieved an average improvement in the micro f1-score of 0.01393 (95% CI $-0.033$ to 0.061), and inward exploration reduced the f1-score by 0.090211 (95% CI 0.037 to 0.143). Detailed results are presented in Tables 1, S5 and S6. In the best settings of parameters (*i.e.*, high p and low q), the resulting network structure representation focuses more on structurally equivalent nodes than on their immediate neighbors, meaning that prediction quality improves when structurally equivalent nodes are taken into account.

**Table 1:** Mean and maximum scores for inward/outward parameters.

	Micro f1-score (%)	Macro f1-score (%)
Neutral	69.4	52.9
Mean outward	70.8	54.8
Mean inward	60.4	40.2
Max outward	71.0	55.1
Max inward	67.5	50.0

**Figure 2:**
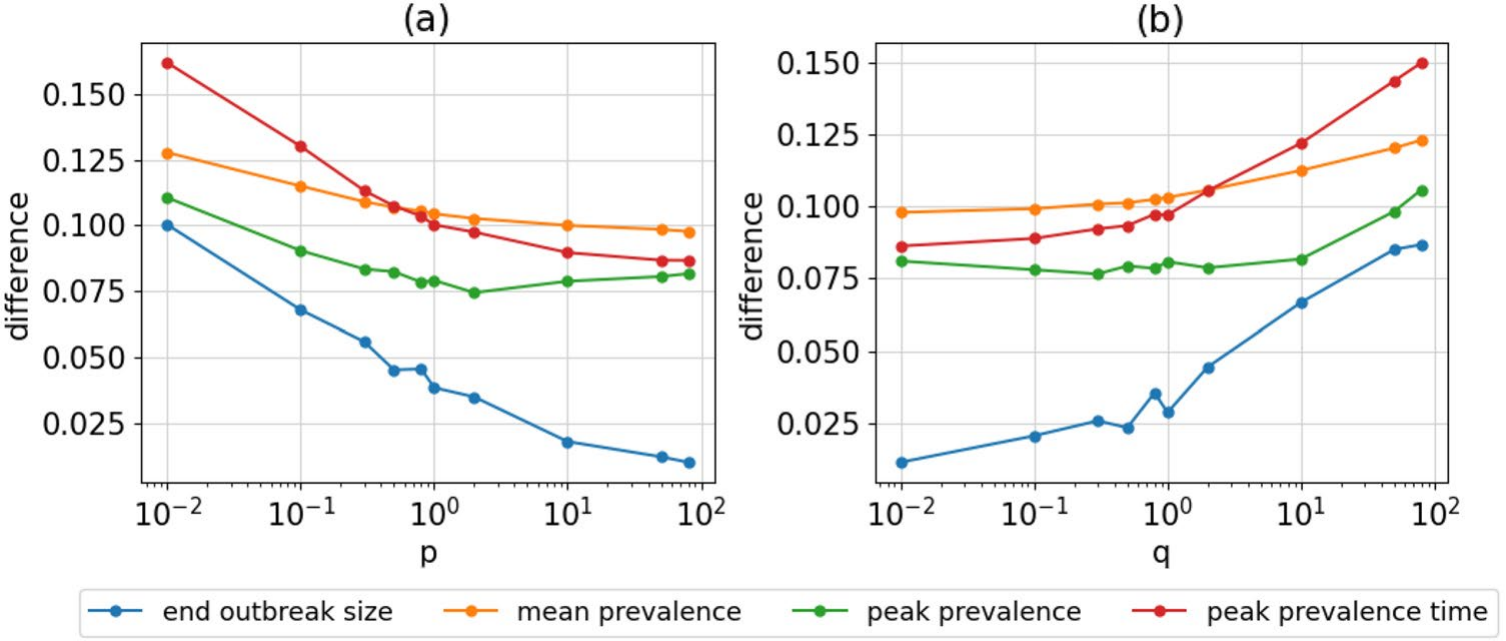
Difference in epidemiological metrics between prediction and simulation.

Additionally, some labels appear less frequently in our ground truth data than others and are therefore harder to predict. This resulted in a lower macro f1-score than the micro f1-score (see Fig. S2). Nevertheless, the prediction accuracy score increases similarly to the micro f1-score by 0.0185 (95% CI −0.029 to 0.066) for outward and decreases by 0.1276 (95% CI 0.091 to 0.164) for inward exploration (see Table 1). With disease-specific metrics, such as outbreak size and prevalence, our results show that outward exploration yields better prediction accuracy than inward exploration. This observation is especially relevant regarding the prediction of final outbreak size and peak prevalence time, where it shows the most significant improvements (see Fig. 2). The prediction of the mean prevalence has the least improvement, with a difference of 0.0284 percentage points (95% CI 0.027 to 0.029). The effect of outward exploration can also be seen in the incidence, with an improvement of 0.0134 percentage points (95% CI 0.011 to 0.015) (see Fig. S5). The improvement of prediction accuracy seems to be the strongest early in the epidemic, showing a higher macro f1-score for the first 10% of time steps (see Fig. S9). This effect is however not reflected in the micro f1-score, where the difference between inward and outward exploration remains the same in different phases of the epidemic (see Fig. S8).

Interestingly, we note a small peak at the neutral values $p=1$ and $q=1$. If either p or q is set to 1, the prediction is either slightly better or worse than what could be expected from the values next to it, suggesting that the two parameters influence each other’s outcomes. This behavior can also be seen in a 3D-plot of the scores for changing p and q, as shown in Fig. S4. This unexpected behavior can only be seen in natural networks, where data were drawn from real subjects, as shown in Fig. S3. We conjecture that this behavior is likely caused by a characteristic of human contact networks that is not well modeled by artificially generated networks^2^. However, even for the real world networks this peak is small, and with outward exploration a more significant improvement can be achieved.

On the modelling and prediction of an infectious disease spreading, two nodes being *similar* means that their label (*i.e.,* predicted outcome at a given time) is more likely to be the same. That is, they will be infected and recover at the same time. Additionally, the state of a node in disease spreading will depend on its neighbors: a node is likely to be infected at the same time as its neighbors as they infect each other. Therefore, knowledge about the state of any neighbouring node can help the prediction accuracy, supporting the importance of considering homophily when studying disease spreading.

In a temporal network, the representation of nodes over time steps may not be ideal for predicting how an infectious disease will spread. As it is typically done, node *i* at time *t* and node *i* at $t+1$ are connected by design of the supra-adjacency network. This representation is closer to reality since these nodes represent the same person at different time steps, *i.e.*, it is likely that they have the same state. However, unlike the task of predicting interests, when predicting the dynamics of an epidemic outbreak, a connection between two nodes is not necessarily caused by their similarity, but rather by both nodes being similar *because* they were in contact. In the corresponding temporal network, this observation results in a time delay, where the two nodes are similar during a single time step after they were in contact.

As shown by our results, structural equivalence can be indicative of the infection time of a person. People who are in the same structural position are likely to be infected around the same time. Additionally, communities might play a significant role in the spread of a disease, where if one community is infected and it has two neighboring communities, then all nodes that act as bridges between these communities are more likely to be infected first, followed by the center nodes in the neighboring clusters, and then the nodes at the edges of these clusters. As a practical result, if we know the state of a node a neighboring cluster, we can then forecast the state for structurally equivalent nodes in the other clusters.

For the process of disease spreading, the balance of structural equivalence and homophily, *i.e.*, of outward and inward exploration in the random walk, allows us to draw conclusions about modelling the disease spreading process. Oftentimes, disease predictions follow the homophily approach – the disease is tracked from one person to the next. However, our results show that the prediction seem to work best with outward oriented parameters, and the prediction of a node’s state is improved by taking into account the information about structurally equivalent nodes in the training set.

## Discussion

Node embeddings are of paramount importance for the study and modelling of any large graph, including the forecast of how an infectious disease may spread. For this purpose, node2vec is able to preserve structural equivalence. This systematic approach allows us to compare the roles homophily and structural equivalence play in large temporal networks of human contacts. Since most infectious diseases, such as COVID-19^8, 24^, require close contact to spread from one person to the next, neighbors in a contact network will help drive how the disease spreads. In other words, the outcome of an outbreak depends on *when* and *how often* nodes are in contact^17, 18^. Additionally, our results support the observation that the position of a person in their contact network seems to be indicative of infection time too.

Based on the results presented in this work, we note that it is possible to improve the prediction of a person’s infection status at a given time by including information about structurally equivalent nodes. This information is encoded in our learning model through the inward and outward exploration parameters of the node embedding algorithm (*i.e.,* node2vec). Better prediction accuracy can help design countermeasures and prevent (or at least slow down) disease spreading. Structural equivalence in a human contact network is closely related to the roles people have in society. On a higher level, this might help identify groups of people that are infected first when only partial information about temporal contacts is available. Particularly, if a high amount of infections is detected within a certain group of people (*e.g.,* staff at hospitals or children at school), it is likely that a structurally equivalent group of people in other similar communities will be infected too.

Furthermore, our results highlight the importance of the availability of temporal contact data to study the spread of infectious diseases. Therefore, in preparation for future pandemics, contact tracing efforts could be established, while still protecting people’s privacy as the highest priority. As human mobility tends to be highly predictable^23^, it may be safe to assume even a sparsely sampled set of contacts prior to an outbreak could be representative. Therefore, yielding strong indicators of an uptick in infections by analyzing structurally similar nodes. Finally, to support and validate this conjecture and our observations, similar analyses are still required using temporal contact data from actual outbreak scenarios instead of simulations.

## Methods

### Structural equivalence and homophily

The goal of a node embedding is to represent each node as a low dimensional feature vector while preserving the structure of the graph. That means that the feature vectors of two similar nodes are similar to each other. Node similarity can be defined differently, and strongly depends on the task. The two most commonly studied similarities between nodes in a human contact network are: (1) *homophily*, defined by the shared neighbors of two nodes, and (2) *structural equivalence*, defined by the resemblance in the structural position any two nodes have in the network.

Often, homophily means that connected nodes are similar to each other as they are likely to share similar contacts. As an example, people with similar interests are more likely to meet as they actively seek other people with the same interests. Therefore, a node embedding that preserves *homophily* would be a good choice if the goal was to predict the interests of people in the network. Note that *homophily* can also be modified to consider 2-hop (or n-hop) neighbors or “friends of friends” as similar, which in a social network, might represent an even stronger predictor than a direct connection^9^. However, as further hops are considered, the expected number of neighbors grows exponentially, thereby reducing the uniqueness of each node alongside the usefulness of the similarity metric.

*Structural equivalence measures the affinity between two nodes based on the similarity of their position in the network*, even though they might not be directly connected. This kind of similarity is often interpreted as a good indicator of the roles people have in society. For example, a manager of a big company would have a different structural position in the contact network than their stay-at-home partner, even though they are in contact frequently.

With *structural equivalence*, two nodes can be considered similar even though they are on different sides of the network, while with *homophily*, similar nodes are always directly next to each other. This makes the two concepts seem opposite, but in human contact networks both may appear at the same time, for example, when a person is similar to their friends, but also to other people in the same role they do not have contact with. For some applications, including disease dynamics prediction, a mixture of the two concepts needs to be preserved and balanced by the embedding algorithm for an accurate representation of society.

### node2vec

node2vec is a node embedding method that allows us to systematically balance *homophily* and *structural equivalence*^10^ through a series of random walks. The number of conducted random walks and the length of these walks are hyperparameters of the embedding algorithm. Additionally, node2vec has two parameters that control the bias in these exploratory random walks. Depending on these parameters, the random walk is more likely to sample nodes that are either far away (*i.e.,* outward exploration) or close to the starting node (*i.e.,* inward exploration). With inward exploration, the random walk becomes more like the search tree of a breadth first search in the graph, while with outward exploration, it is more similar to a depth first search.

The return parameter p controls the probability with which the random walk returns to the last visited node. A high p means the random walk is less likely to return, and a low p results in nodes that are already in the random walk being sampled more often, yielding even higher similarities between those nodes. To balance this effect, the in-out parameter q allows the random walk to favor nodes that lie further away from the starting node. With low q, the random walk is biased towards outward lying nodes, specifically, the random walk reaches a greater variety of nodes and those further away can still be considered similar. The authors of the original node2vec paper^10^ propose that inward exploration preserves *homophily* in the network, while outward exploration preserves *structural equivalence*.

All temporal networks were embedded with node2vec using different hyperparameters (see Table S4). Our experiments have shown that the random walk length and the number of random walks do not have much influence on the results, so we fixed those parameters to 10 random walks of size 80. Since p and q are positive and 1 is the neutral value, we distributed the values for p and q logarithmically between 0 and 80. We tested 10 different values for each parameter, 5 for inward and 5 for outward exploration. These runs resulted in 100 different possible pairings for p and q. All of these embeddings were then used to predict the labels gained from 250 SIR-simulations for each network. The prediction algorithm used was logistic regression with l2 regularization.

### Datasets

We used 24 datasets, of which 6 are derived from real world data. Five of these datasets were collected between the years 2009 and 2015 at several locations, each collection campaign lasting between 2 days and 2 weeks. In all cases, data were sampled anonymously in order to preserve the identity of the subjects. Furthermore, all subjects or their legal guardians provided explicit **informed consent** in having their data collected and analyzed. The Reality Mining dataset^3^ was collected with explicit consent from all subjects by the MIT Human Dynamics Lab, and the use of the set was authorized for such study as long as the privacy of the participants was protected (*i.e.*, no de-anonymization was attempted). The remaining datasets include temporal networks, collected with the consent of the subjects or someone responsible for them^7^. In our analyses, all methods were performed in accordance with the relevant ethical and legal guidelines and regulations.

The empirical data from those datasets were sampled with the same method: a setup of RFID scanners at fixed locations and wearable devices for each subject. A contact was documented whenever two participants were registered by the same set of readers at the same time. In the datasets, all contacts are listed by the IDs of the participants and the corresponding time step with a resolution of 20 seconds^7^. Only one dataset, the Reality Mining dataset, used contacts detected by Bluetooth scans on smartphones. 100 students and faculty members of the MIT Media Laboratory participated in this study, that took place over 9 months in 2004. The scans were conducted every 5 minutes and included timestamps in seconds^3^. Detailed information about the datasets can be found in Table S2. From these data, we derived temporal networks with one node for each participant at each time step and edges as contacts. Data were aggregated to time steps of 10 minutes, a time window that achieves a good balance between the minimal contact duration necessary for a disease to spread and the number of time steps to be analyzed in this study. The other 18 networks were artificially created models of human contact networks. In 9 of them the nodes are connected randomly and the node degree distribution is binomial. In the other 9 networks, the node degree is power-law distributed, which is closer to what has been found in networks derived from real world data^20^.

### SIR simulation

As reference for how a disease spreads in these networks, a dynamic SIR simulation was conducted. In an SIR simulation, a random node in the network is infected. Then, at each time step, an infected node infects one of its neighbors with probability $\alpha$ (infection rate) and recovers with probability $\mu$ (recovery rate). This provides each node at each time step with one of three labels: *susceptible* (S), *infected* (I) or *recovered* (R). In this simplified model of disease progression, “infected” means that a person is contagious, “susceptible” means that a person can become infected and “recovered” means that a person cannot become infected and does not play a role in the disease spreading at this time. In an SIR simulation, the nodes always follow the same disease progression from S to I then R. Note however, that the prediction model is agnostic about the semantics or the order of these labels, and it could just as well predict SIS or SI simulations. We conducted simulations with 5 different parameter sets, which can be found in Table S4.

### Network representation

To prepare the dynamic networks for the prediction, the time steps of each network were connected into a static supra-adjacency network. Nodes can be identified by the pair (k, t), where k is the person this node represents and t the current time step. If a node is infected at time step t, it is likely to still be infected at $t+1$. To use this temporal dependency in the prediction, the time steps of the network are interconnected. This interconnection is done by making node (i, t) always connected to (i, t+1). Furthermore, if there is a contact between person i and person j at time t, there exists an edge from (i, t) to (j, t+1) and from (j, t) to (j, t+1). Additionally, in order to reduce the number of nodes that need to be embedded, only active nodes are considered. These are nodes (i, t) where i had at least one contact at time step t. Inactive nodes are deleted and their incoming edges are rerouted to their next active future self, as previously done by Sato *et al.*^21^. Finally, we converted all 24 datasets into supra-adjacency networks as described. Table S3 shows the different sizes and densities of the real world networks, and Table S1 summarizes those of the artificial ones. In prediction, some of the networks performed better than others, but all showed the described improvements for outward exploration (see Figs. S1, S6, S7 and Table S7).

### Evaluation metrics

We primarily chose the f1-score as an evaluation metric for its robustness in evaluating prediction accuracy, by balancing true and false positives or negatives. Since the size of classes can be strongly imbalanced, we look at two different versions to expand the f1-score to multiple labels, namely the *micro f1-score*, which weighs all classes the same, and the *macro f1-score*, that evaluates the score for each class separately and reports the average of all classes. Additionally, we considered different disease-specific metrics that are related to its spreading process:

- End outbreak size, or the number of people that are infected or recovered at the end of the simulation. This number expresses how many people were directly affected by the epidemic.
- Mean prevalence, or the average number of infected people per time step, which indicates the expected outbreak size at any time step.
- Peak prevalence, or the maximum number of people that were infected at any time step. This metric is relevant to estimate the capacity hospitals need to have to provide care for infected people in the worst time of the epidemic outbreak.
- Peak prevalence time, which is the time step when the peak prevalence occurs and is often associated with how aggressively the disease spreads.
- Mean incidence, or the average number of nodes that changed their state from susceptible to infected in one time step. It is indicative of the rate with which the disease spreads.

All of these metrics are evaluated as the difference between simulation and prediction. To meaningfully compare results between different networks, they are given as percentage of participants in the network or percentage of the number of time steps.

## Supplementary Information

Supplementary Information.

## Data Availability

The Reality Mining set^3^ can be requested from the MIT Human Dynamics Lab at http://realitycommons.media.mit.edu/realitymining4.html. All other contact data^7^ can be found at https://doi.org/10.5281/zenodo.2540795. The artificial networks were created with networkX^11^.
