# Supplementary material for: On the importance of structural equivalence in temporal networks for epidemic forecasting
Source: Sci Rep. 2023 Jan 17;13:866. doi: 10.1038/s41598-023-28126-w (PMC9843108; doi:10.1038/s41598-023-28126-w)
Supplement: Supplementary file 1 — Supplementary Information. [file 41598_2023_28126_MOESM1_ESM.pdf]

On the importance of structural equivalence in temporal networks for epidemic forecasting
Supplementary Information

Pauline Kister[1], Leonardo Tonetto[1]*

[1]Technical University of Munich
November 16, 2022

Supplementary Information

distribution	$\lvert V_A \rvert$	$\lvert V \rvert$	$\lvert T_A \rvert$	$\lvert T \rvert$	$\lvert E \rvert$
binomial	44925	1000	100	100	29912
binomial	45112	1000	100	100	29998
binomial	45105	1000	100	100	29951
binomial	17926	100	400	400	12080
binomial	17395	100	399	400	11644
binomial	17443	100	400	400	11718
binomial	2116	10	275	300	2281
binomial	2233	10	290	300	2476
binomial	2050	10	286	300	2154
powerlaw	45057	1000	100	100	29913
powerlaw	45149	1000	100	100	30003
powerlaw	45027	1000	100	100	29956
powerlaw	17879	100	400	400	12076
powerlaw	17501	100	399	400	11646
powerlaw	17390	100	400	400	11709
powerlaw	2110	10	275	300	2256
powerlaw	2209	10	290	300	2493
powerlaw	2060	10	286	300	2142

Table S1: Artificial Networks: node degree distribution, $|V_A|$ active nodes, $|V|$ participants, $|T_A|$ active timesteps, $|T|$ timesteps and $|E|$ number of edges

name	place	year	participants	duration	sampling method
InVS15	work office	2015	232	2 weeks	RFID
LH10	hospital	2010	81	3 days	RFID
LyonSchool	primary school	2009	242	2 days	RFID
realitymining	university	2004	100	9 months	Bluetooth
SFHH	conference	2009	403	2 days	RFID
Thiers13	high school	2013	326	1 week	RFID

Table S2: Metadata of the data sets

network	$\lvert V_A \rvert$	$\lvert V \rvert$	$\lvert T_A \rvert$	$\lvert T \rvert$	$\lvert E \rvert$
InVS15	22133	217	698	1656	18386
LH10	5219	76	359	477	7580
LyonSchool	18641	242	116	217	45008
realitymining	21613	102	980	1025	50897
SFHH	10693	403	128	191	17003
Thiers13	32015	327	246	606	34920

Table S3: Metadata of the dynamic networks: $|V_A|$ active nodes, $|V|$ participants, $|T_A|$ active timesteps, $|T|$ timesteps and $|E|$ number of edges

(infection rate, recovery rate)
(0.13, 0.002)
(0.25, 0.002)
(0.13, 0.005)
(0.25, 0.005)
(0.25, 0.007)

Table S4: Parameters for the SIR simulations

q \ p	0.01	0.3	0.5	1.0	10.0	50.0	80.0
0.01	68.60%	70.65%	70.97%	70.80%	70.82%	71.01%	70.93%
0.3	63.65%	67.67%	68.75%	70.29%	69.02%	70.40%	70.33%
0.5	60.93%	69.27%	70.05%	70.08%	70.51%	70.43%	70.73%
1.0	60.46%	67.66%	68.95%	69.74%	70.15%	69.41%	70.44%
10.0	55.19%	57.96%	62.22%	64.09%	65.51%	67.09%	67.44%
50.0	58.03%	55.86%	57.56%	58.81%	60.32%	61.53%	64.33%
80.0	58.13%	56.36%	57.26%	60.29%	59.12%	60.93%	62.43%

Table S5: Average micro f1-score for different values of `p` and `q`, with outward exploration marked in pink and inward exploration in white

q \ p	0.01	0.3	0.5	1.0	10.0	50.0	80.0
0.01	-1.15%	0.91%	1.22%	1.06%	1.08%	1.26%	1.18%
0.3	-6.09%	-2.07%	-0.99%	0.55%	-0.73%	0.66%	0.58%
0.5	-8.81%	-0.47%	0.31%	0.34%	0.77%	0.69%	0.99%
1.0	-9.28%	-2.08%	-0.79%	0.00%	0.40%	-0.34%	0.69%
10.0	-14.55%	-11.78%	-7.53%	-5.65%	-4.23%	-2.65%	-2.30%
50.0	-11.72%	-13.88%	-12.18%	-10.94%	-9.42%	-8.22%	-5.41%
80.0	-11.62%	-13.38%	-12.48%	-9.45%	-10.62%	-8.82%	-7.31%

Table S6: Difference in micro f1-score to the unbiased embedding

score \ network	InVS15	LH10	LyonSchool	realitymining	SFHH	Thiers13
outward micro	0.6678	0.7825	0.8679	0.6546	0.8166	0.7627
neutral micro	0.6415	0.7678	0.8505	0.6380	0.7810	0.6541
inward micro	0.5132	0.7114	0.7421	0.5852	0.6910	0.6023
outward macro	0.5894	0.5441	0.6079	0.5482	0.5626	0.5690
neutral macro	0.5595	0.5104	0.5897	0.5099	0.5308	0.4555
inward macro	0.3971	0.4109	0.4705	0.4022	0.4525	0.4013

Table S7: average f1-scores for different networks and inward/outward oriented random walks

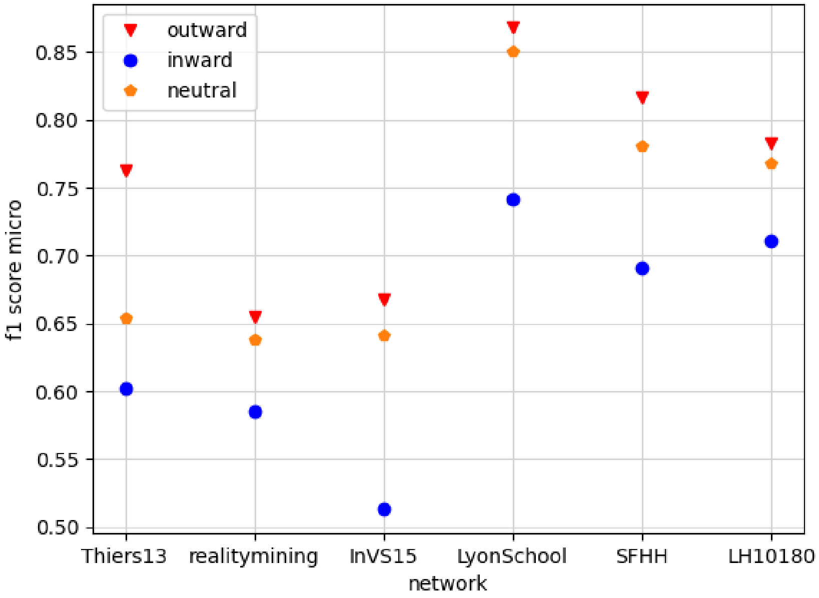

Figure S1: Inward, outward and neutral p and q for individual networks

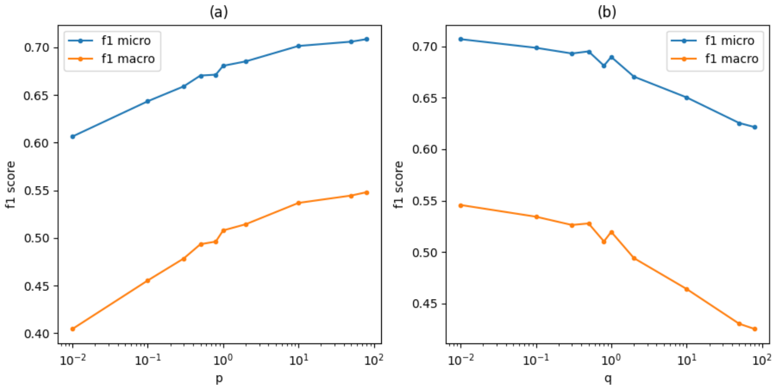

Figure S2: Comparisons of micro and macro f1-score for different values of p and q

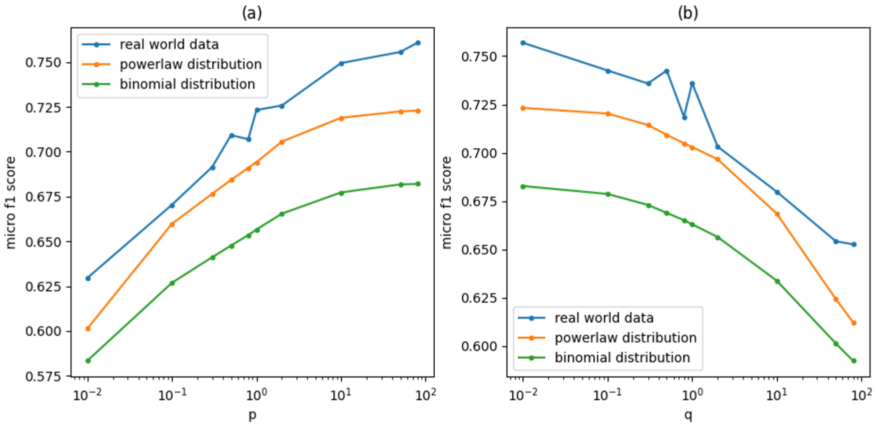

Figure S3: Comparison of the micro f1-score for different distributions of node degree

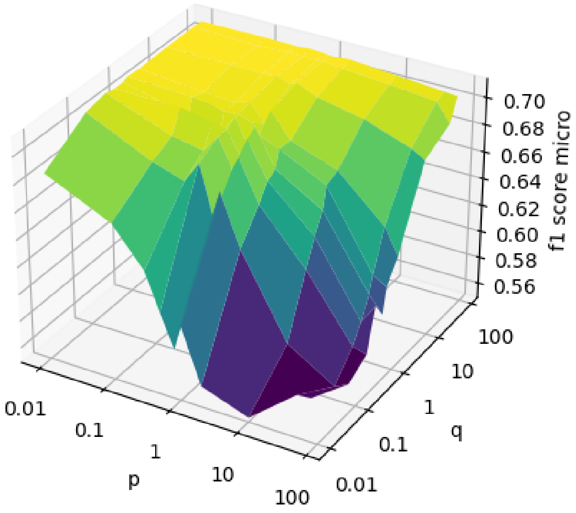

Figure S4: micro f1-score surface plot

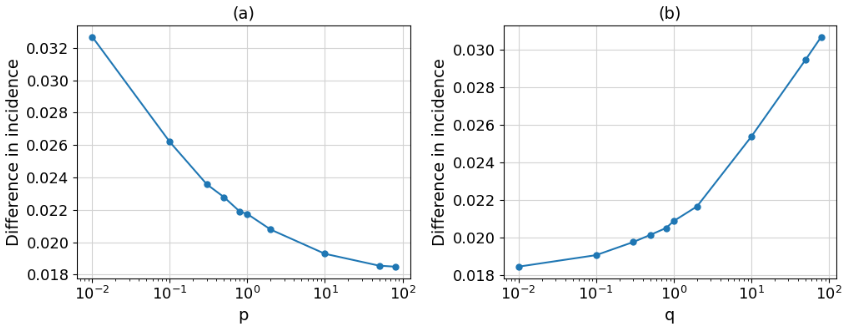

Figure S5: Comparisons of the mean incidence for different values of p and q

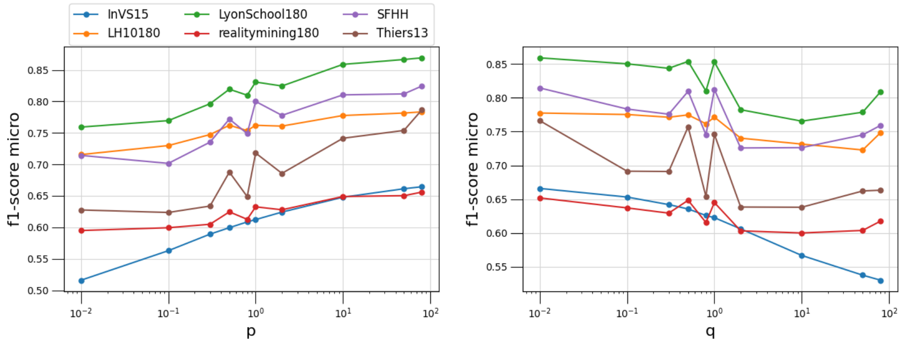

Figure S6: Comparison of the micro f1-scores of real-world networks for different values of p and q

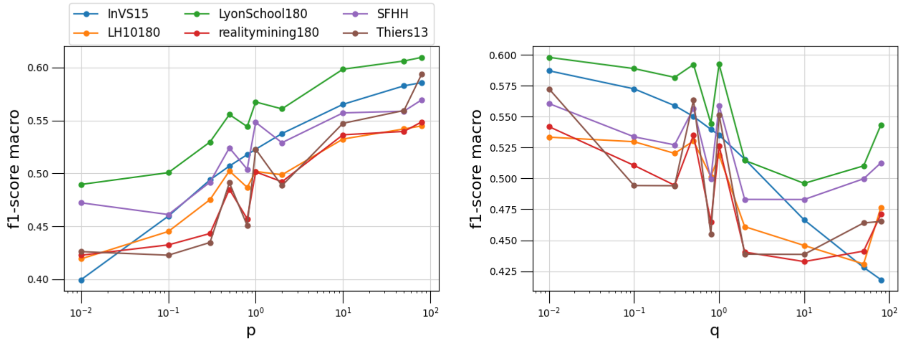

Figure S7: Comparison of the macro f1-scores of real-world networks for different values of p and q

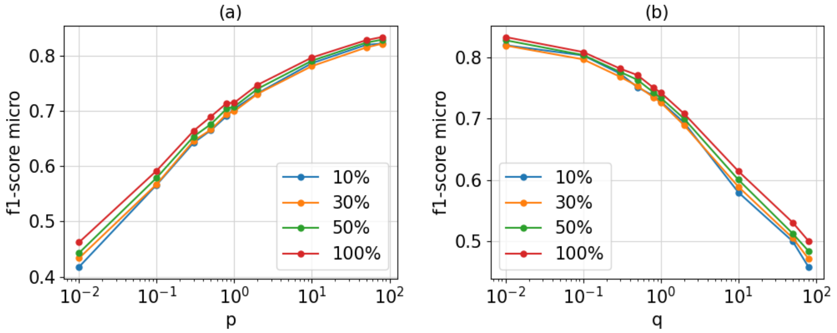

Figure S8: Comparisons of micro f1-scores for the first 10%, 30% and 50% of time steps for a random sample of 150000 predictions

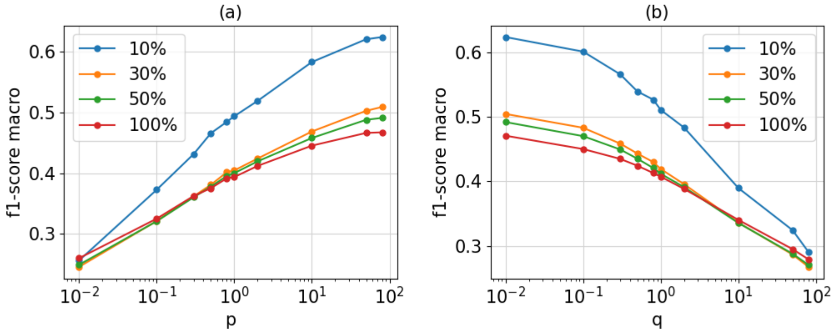

Figure S9: Comparisons of micro f1-scores for the first 10%, 30% and 50% of time steps for a random sample of 150000 predictions
